# Supplementary figures and images for: Effects of Restricted Feeding on Growth Performance, Intestinal Immunity, and Skeletal Muscle Development in New Zealand Rabbits
Source: Animals (Basel). 2022 Jan 10;12(2):160. doi: 10.3390/ani12020160 (PMC8772555; doi:10.3390/ani12020160)

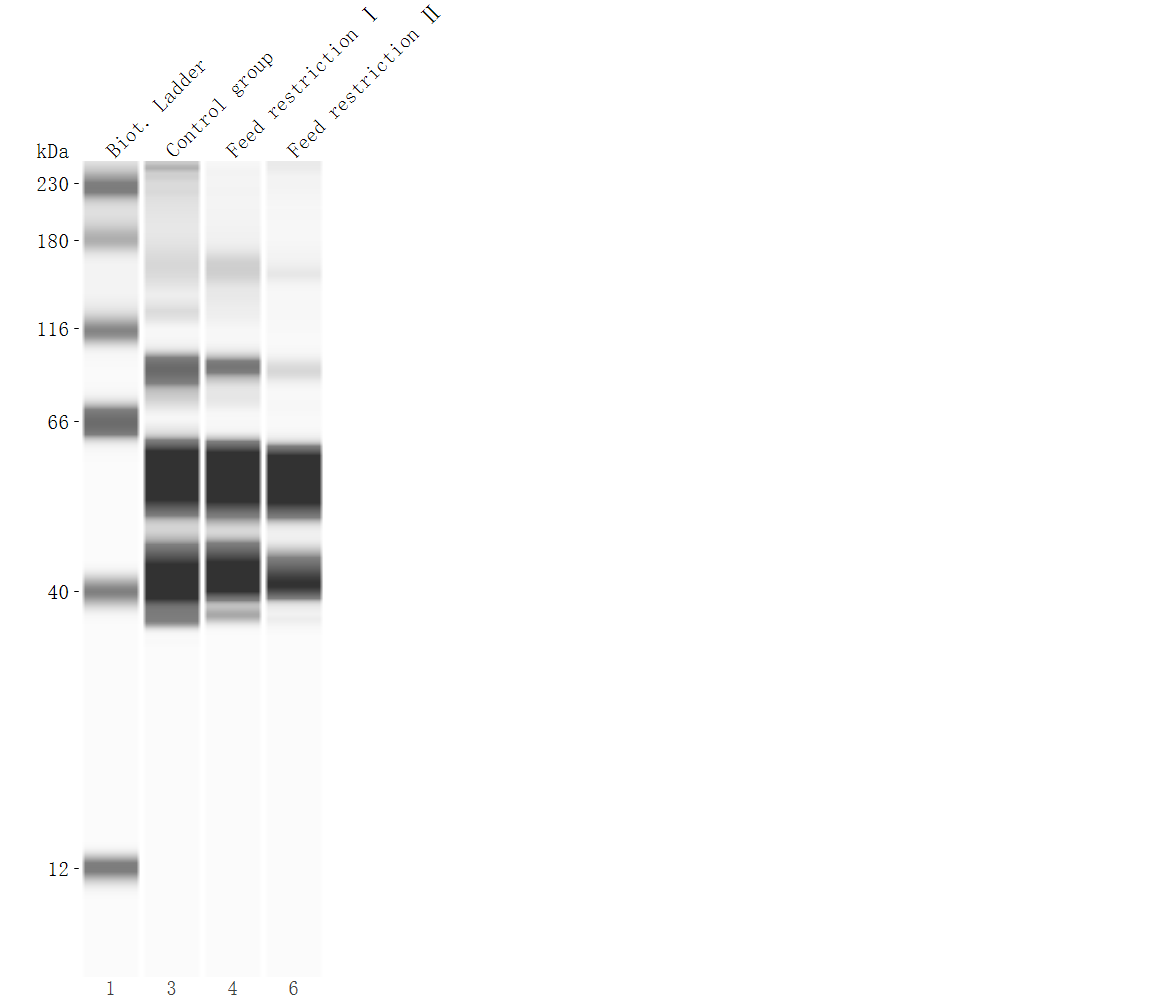

Supplement: Supplementary file 1 [file animals-12-00160-s001.zip › Supplementary Materials/Figure S1. Western Blot Figure for AKT..png]

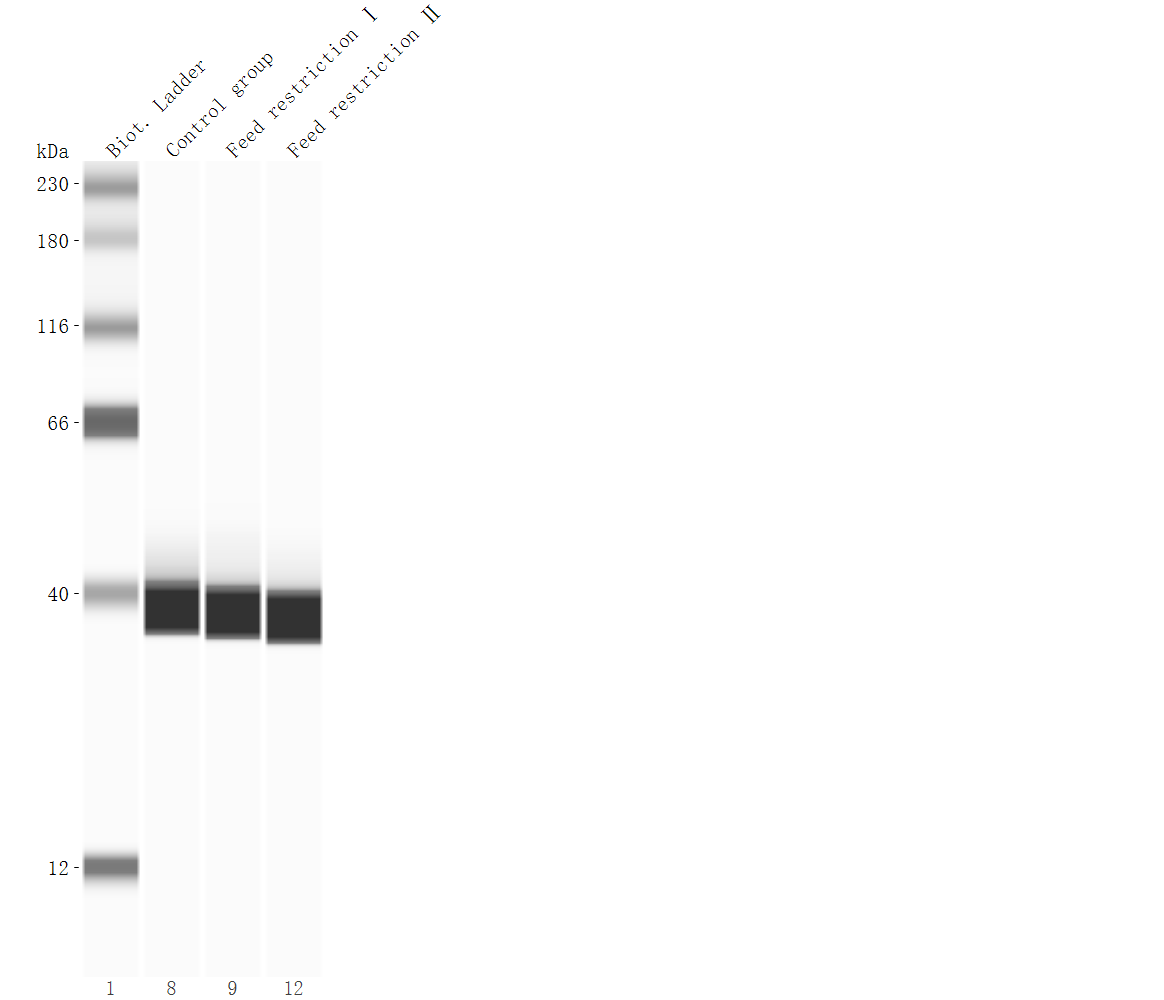

Supplement: Supplementary file 1 [file animals-12-00160-s001.zip › Supplementary Materials/Figure S2. Western Blot Figure for GAPDH..png]

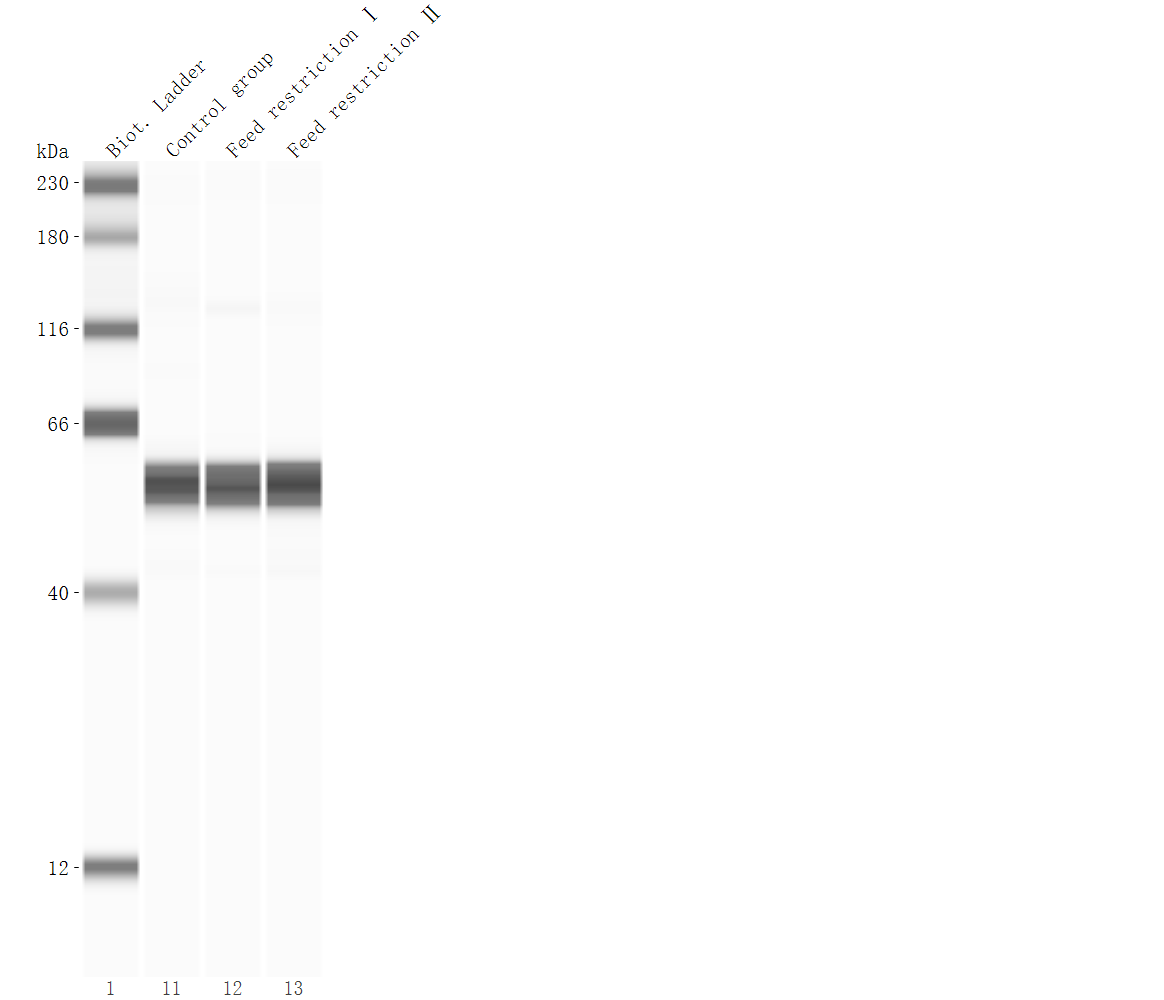

Supplement: Supplementary file 1 [file animals-12-00160-s001.zip › Supplementary Materials/Figure S3. Western Blot Figure for p70..png]

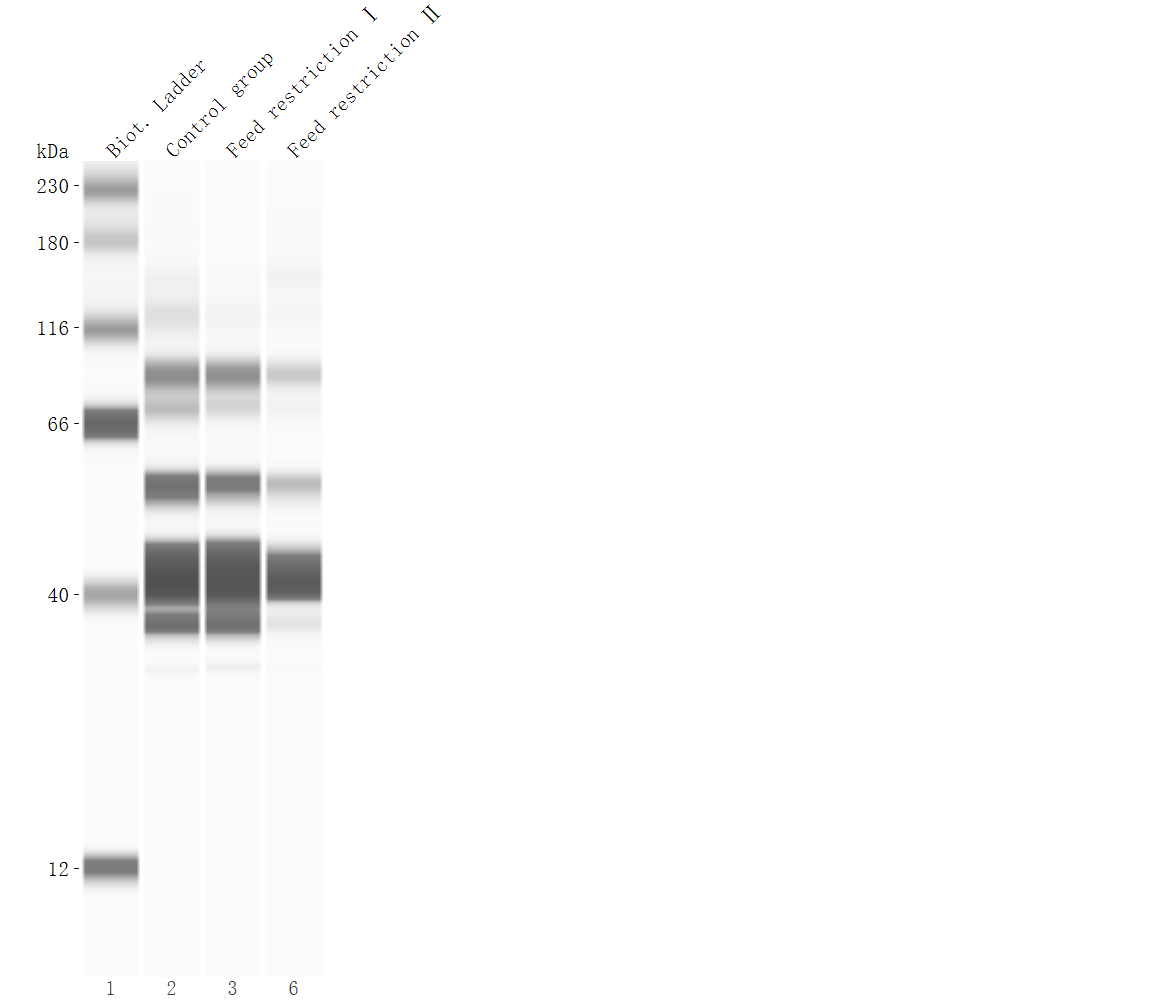

Supplement: Supplementary file 1 [file animals-12-00160-s001.zip › Supplementary Materials/Figure S4. Western Blot Figure for p-akt..png]

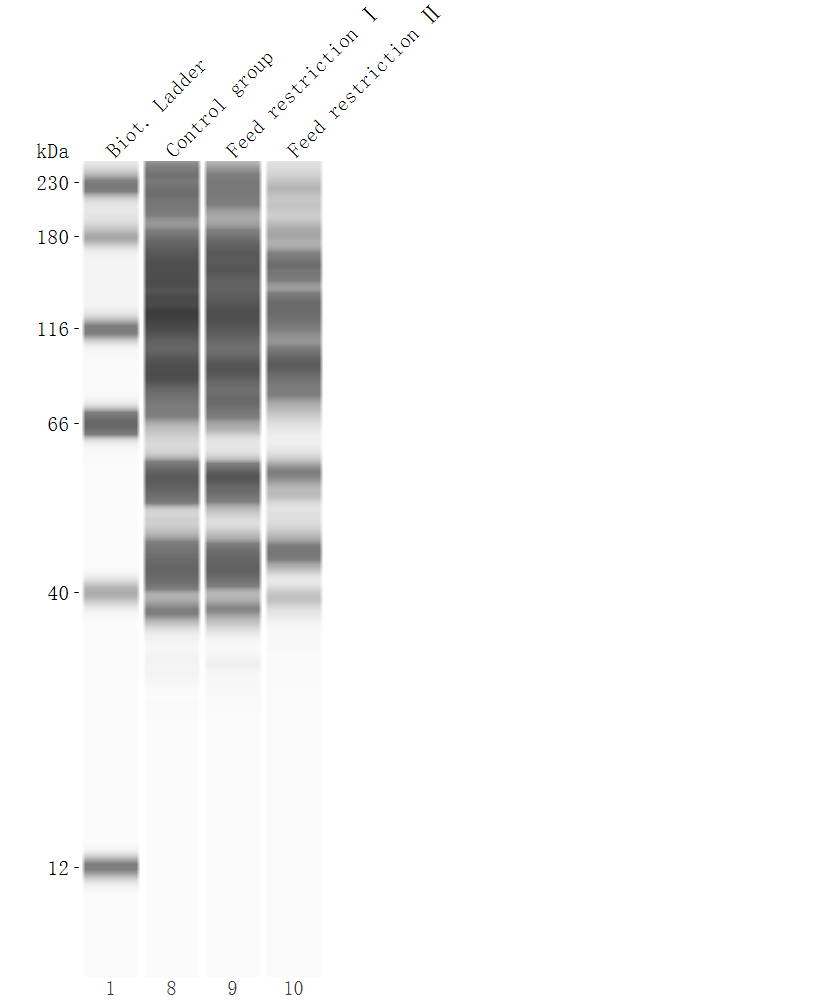

Supplement: Supplementary file 1 [file animals-12-00160-s001.zip › Supplementary Materials/Figure S5. Western Blot Figure for p-p70..png]
